# Molecular cloning, characterization and 3D modelling of spotted snakehead *fbn1* C-terminal region encoding asprosin and expression analysis of *fbn1*

**DOI:** 10.1038/s41598-023-31271-x

**Published:** 2023-03-18

**Authors:** Priyanka Sathoria, Bhawna Chuphal, Umesh Rai, Brototi Roy

**Affiliations:** 1 Department of Zoology, Maitreyi College, University of Delhi, Chanakyapuri, Delhi, 110021 India; 2 Department of Zoology, University of Delhi, Delhi, 110007 India; 3 University of Jammu, Jammu, Jammu and Kashmir 180006, India

**Keywords:** Computational biology and bioinformatics, Molecular biology, Zoology, Endocrinology

## Abstract

The *FBN1* gene encodes profibrillin protein that is cleaved by the enzyme furin to release fibrillin-1 and a glucogenic hormone, asprosin. Asprosin is implicated in diverse metabolic functions as well as pathological conditions in mammals. However, till date, there are no studies on asprosin in any non-mammalian vertebrate. In this study, we have retrieved the spotted snakehead *Channa punctata fbn1* gene (ss *fbn1*) from the testicular transcriptome data and validated it. The transcript is predicted to encode 2817 amino acid long putative profibrillin protein. Amino acid sequence alignment of deduced ss profibrillin with human profibrillin revealed that the furin cleavage site in profibrillin is well conserved in *C. punctata*. Further, differential expression of ss *fbn1* was observed in various tissues with the highest expression in gonads. Prominent expression of *furin* was also observed in the gonads suggesting the possibility of proteolytic cleavage of profibrillin protein and secretion of asprosin in *C. punctata*. In addition, the C-terminal of the *fbn1* gene of *C. punctata* that codes for asprosin protein has been cloned. Using in silico approach, physicochemical properties of the putative ss asprosin were characterized and post-translational changes were predicted. The putative ss asprosin protein sequence is predicted to consist of 142 amino acid residues, with conserved glycosylation sites. Further, the 3D model of ss asprosin was predicted followed by MD (molecular dynamics) simulation for energy minimization. Thus, the current study, for the first time in non-mammalian vertebrates, predicts and characterizes the novel protein asprosin using in silico approach.

## Introduction

The *FBN1 *gene in mammals is reported to encode profibrillin protein that is cleaved by activated protease, furin into fibrillin-1 and 140-amino acids-long asprosin^1^. Furin is a proprotein convertase belonging to a family of subtilisin serine proteases that cleaves at the R-X-K/R-R↓ or R/K-X-X-X-K/R-R↓ motif in the target protein and is ubiquitously expressed^2^. Fibrillin-1 is a major glycoprotein of extracellular matrix, belonging to the fibrillin family of proteins^3^. The C-terminal region of *FBN1*, specifically exons 65 and 66 are responsible for encoding the glucogenic hormone asprosin that was discovered by Romere et al. in 2016^1^.

Asprosin is majorly expressed in the white adipose tissue in mammals^1^. It is shown to have diverse physiological effects acting through different receptors. This metabolic hormone regulates glucose release from the liver through interaction with G-protein coupled receptor (GPCR), olfactory receptor 734 (*OLFR734*)^4^. It also regulates the orexigenic effect via acting on the agouti-related peptide (AgRP) neurons in the brain^5^. In addition to this, asprosin regulates insulin secretion and inflammation in pancreas^6^. Not surprisingly, asprosin is implicated in various metabolic disorders including type 2 diabetes^7^, obesity^8,9^ and polycystic ovary syndrome^10,11^. In recent years, the profound effects of asprosin on reproductive functions have also been demonstrated^12–15^. However, all these studies are confined to mammals and till date the existence of asprosin in non-mammalian vertebrates is obscure.

In view of this lacuna, the present study was undertaken in fish *Channa punctata*. Teleost are the oldest and most diverse extant vertebrates. In this study, using various bioinformatics tools, *fbn1* and *furin* genes were validated and the furin cleavage site was determined in the *fbn1* encoded putative profibrillin protein in spotted snakehead. Further, tissue-dependent differential expression of the *fbn1* and *furin* gene was demonstrated. In addition, an attempt has been made to overexpress ss asprosin in a heterologous bacterial system. Using in silico approach, the physicochemical properties and post-translational modifications have been predicted for ss asprosin. The 3D modelling of the deduced ss asprosin was carried out using I-TASSER followed by energy minimization through MD simulation.

## Results

### Identification and validation of *fbn1* and *furin* sequence

The potential longest transcript of *fbn1* obtained from testicular transcriptome data of *C. punctata* contained 8454 base pair long coding sequence and was partial from the 5′-end. Similarly, the best transcript of *C. punctata furin* containing 1869 base pair open reading frame (ORF) was retrieved and it was partial from the 5′-end.

### Expression analysis of *fbn1* and *furin* gene in *Channa punctata*

**Figure 1:**
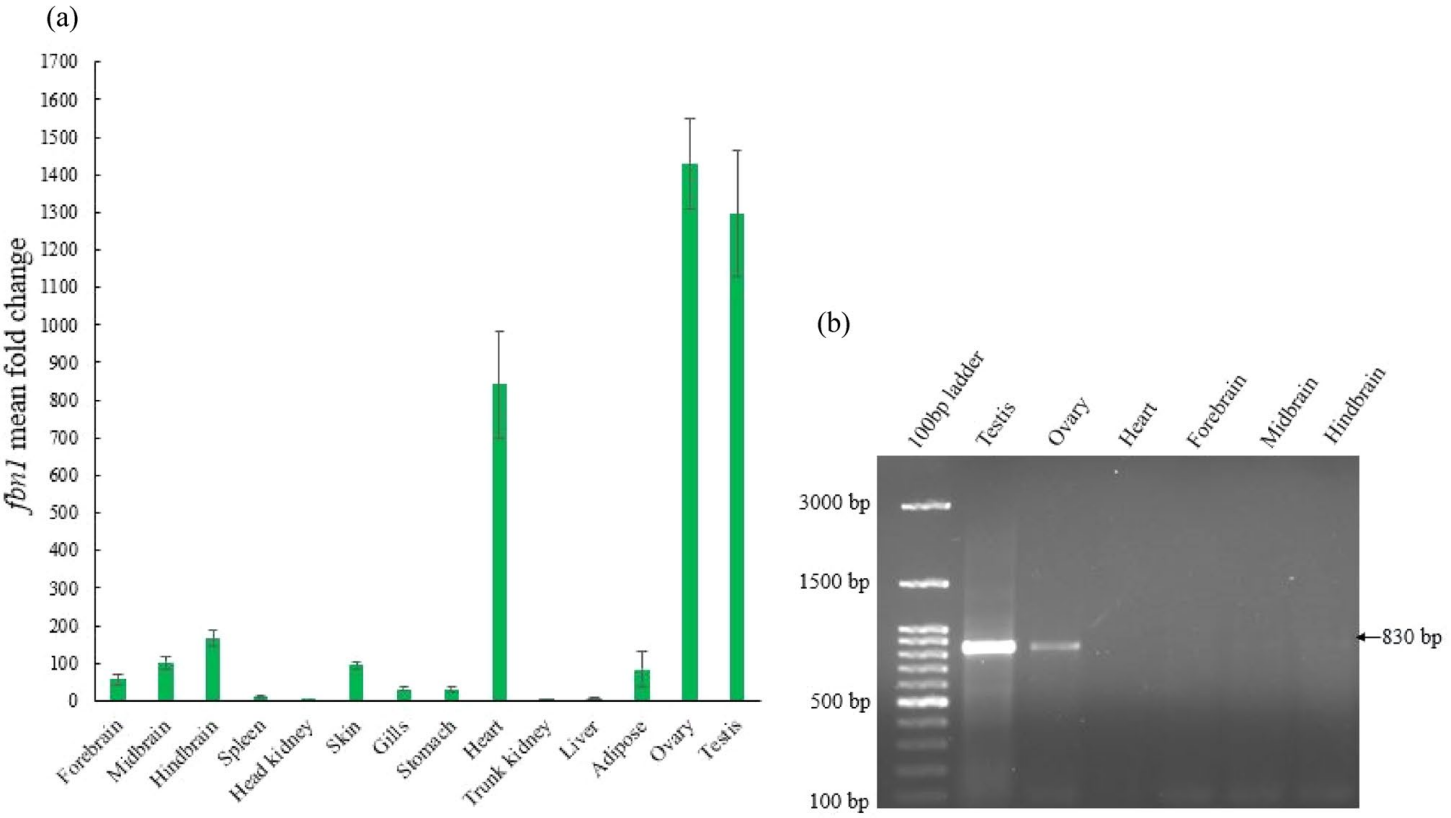
(**a**) Tissue distribution of *fbn1* gene in different tissues of *C. punctata*. Real-time quantitative PCR was used to quantify the gene expression in each tissue. Each data point represents the mean ± SEM of tissues collected from 6 fish (N = 6). Two technical replicates for each sample were used. (**b**) Tissue distribution of *furin* using semi quantitative PCR. The gene expression of *furin* was studied in tissues which exhibited prominent *fbn1* expression (testis, ovary, heart, forebrain midbrain and hindbrain) using semi-quantitative PCR followed by resolving into the 1% agarose gel.

The expression analysis of *fbn1* in different tissues of *C. punctata* revealed the highest expression in gonads followed by heart and different parts of the brain. Among the tissues that expressed *fbn1* prominently (testis, ovaries, heart and brain), *furin* was highly expressed in gonads as observed by semi-quantitative PCR (Fig. 1; Supplementary Fig. S1).

### Identification of furin cleavage site

**Figure 2:**
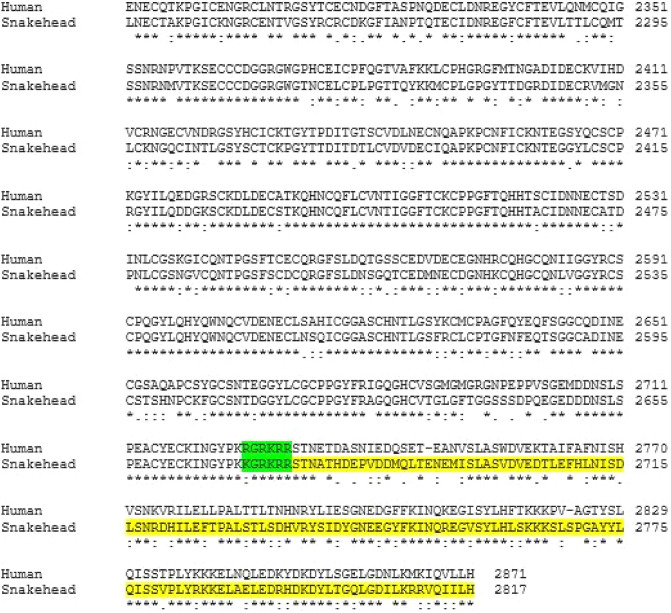
Amino acid alignment of C-terminal region of profibrillin of human and snakehead. The furin cleavage site has been highlighted in green colour and the predicted mature ss asprosin has been highlighted in yellow colour. The ‘*’ represents amino acids that are conserved, ‘:’ indicates amino acids with strongly similar properties, ‘.’ represents amino acids with less similar properties whereas the gaps represent mismatch residues.

The ss *fbn1* transcript encoded 2817 amino acid long putative protein. The multiple sequence alignment of deduced profibrillin of *C. punctata* with profibrillin of *H. sapiens* showed the conserved furin cleavage site (R/K-X-X-X-K/R-R↓) (Fig. 2). On the basis of the presence of furin cleavage site, amino acid sequence of asprosin protein was deduced in *C. punctata*.

### Cloning and characterization of asprosin

#### Cloning and expression of ss recombinant asprosin

**Figure 3:**
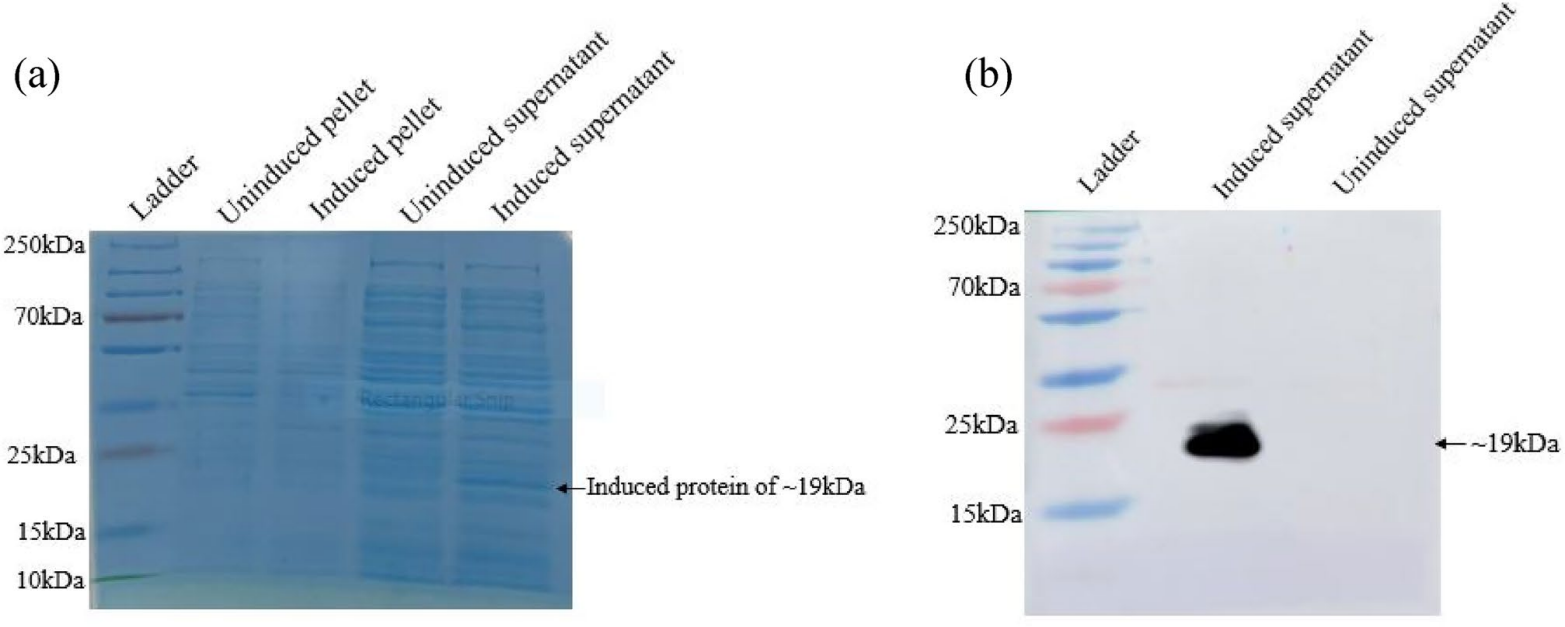
Expression analysis of recombinant ss asprosin. (**a**) 15% SDS-PAGE showing the protein expression in the uninduced pellet, induced pellet, uninduced supernatant and induced supernatant samples observed using coomassie staining. (**b**) Western blotting using anti-His antibody for validation of recombinant asprosin in supernatant of induced and uninduced samples.

The 429 base pair sequence of *fbn1* gene with one stop codon was cloned in the pProEx-HTc vector, transfected into DH5ɑ and validated through sequencing. After transformation in the BL21 cells, induction using IPTG was carried out. Proteins isolated from the uninduced pellet, induced pellet, uninduced supernatant and induced supernatant samples were analyzed using coomassie staining for presence of recombinant asprosin protein (Supplementary Fig. S2). Significant expression of recombinant asprosin (~ 19 kDa) having six histidine tag was observed in the supernatant of induced sample as compared to uninduced supernatant sample. However, in case of pellet, no protein band was detected around 19 kDa in both uninduced as well as induced sample. Further, using anti-His antibody, recombinant asprosin protein with histidine tag was validated in the induced supernatant sample (Fig. 3, Supplementary Fig. S3).

#### Physicochemical properties

**Figure 4:**
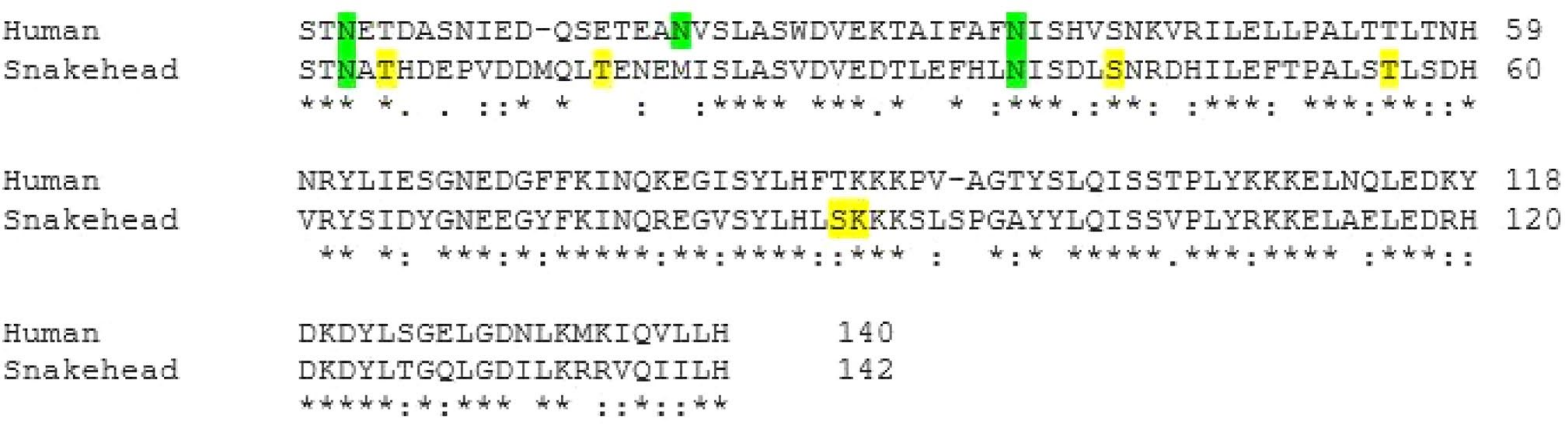
Sequence alignment of putative ss asprosin and human asprosin showing post-translational modifications. The glycosylation sites are highlighted in green and phosphorylation sites are highlighted in yellow.

The putative ss asprosin and human asprosin comprises 142 and 140 residues, respectively with approximately 16 kDa molecular weight. The instability index of putative ss asprosin was 33.89 and human asprosin was 37.84. The aliphatic index of the putative ss asprosin and human asprosin was 94.72 and 89.86, respectively. The GRAVY values of putative ss asprosin was -0.585 and human asprosin was − 0.549 (Table 1). The percent identity matrix of the primary sequence of putative asprosin of *C. punctata* with asprosin of *H. sapiens* revealed 57.86% similarity. The post-translational modifications such as N-linked glycosylation and phosphorylation were predicted using Motif scan software (Fig. 4). Two conserved N-linked glycosylation sites at position 3 and 37 were predicted in ss asprosin. Phosphorylation sites at 5, 16, 42, 56, 88 and 89 positions were predicted in asprosin of *C. punctata*.

**Table 1:** Physicochemical properties of asprosin protein using ExPASy ProtParam tool.

Physicochemical properties	*H. sapiens*	*C. punctata*
Number of amino acids	140	142
Molecular weight (in kDa)	15.88	16.32
Total number of negatively charged residues (Asp + Glu)	20 (14.3%)	25 (17.6%)
Total number of positively charged residues (Arg + Lys)	16 (11.43%)	15 (10.56%)
Instability Index	37.84	33.89
Aliphatic Index	89.86	94.72
Grand average of hydropathicity (GRAVY)	− 0.549	− 0.585

#### Protein modelling, quality assessment and validation of asprosin

**Figure 5:**
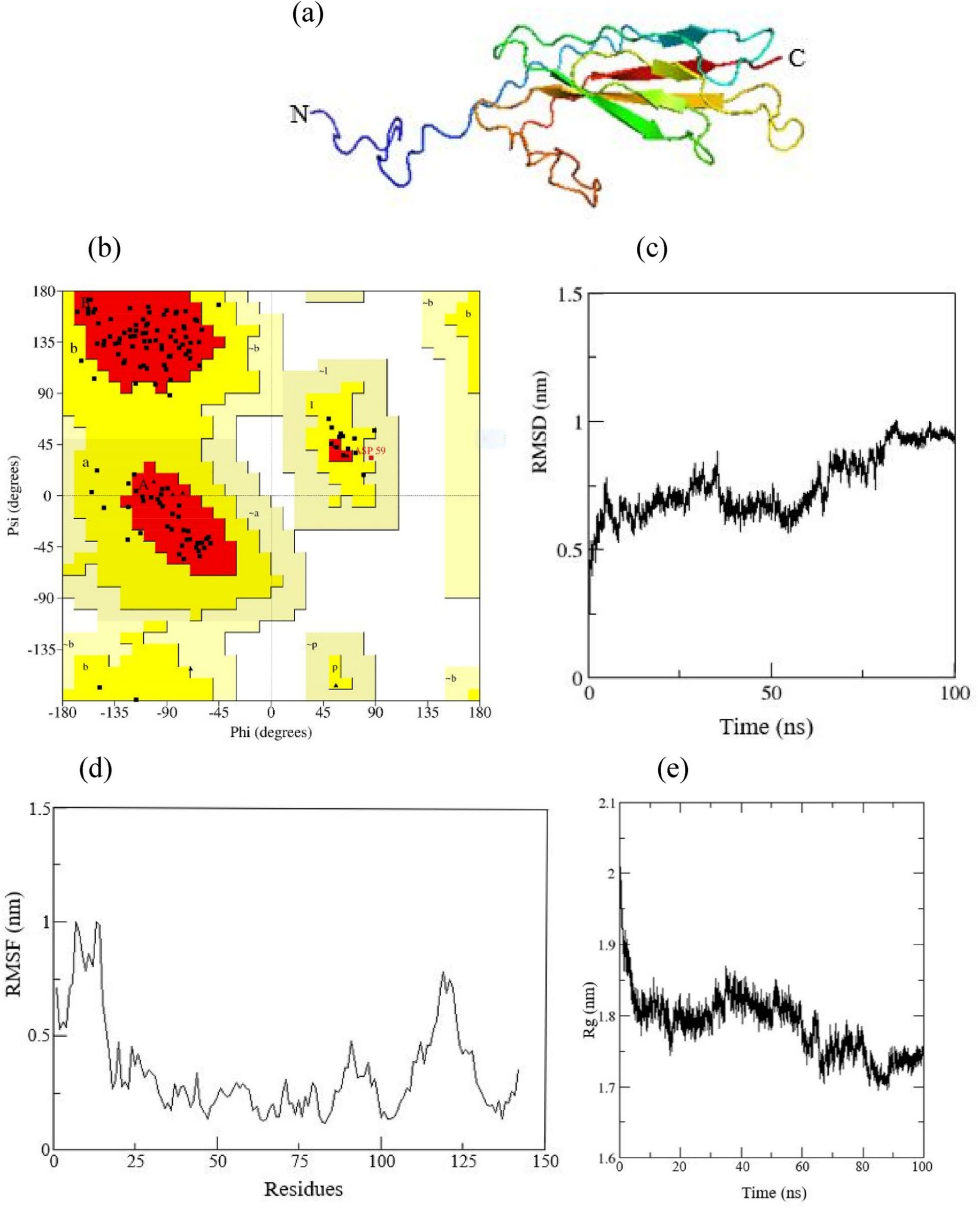
Structural representation and stability parameters of ss asprosin after stimulation. (**a**) 3D model of ss asprosin in which arrow represent the β-sheets. (**b**) Ramachandran plot of amino acid residues of ss asprosin. (**c**) RMSD (Root mean square deviation) of the backbone Cɑ atoms of ss asprosin vs time. (**d**) RMSF (Root mean square fluctuation) of each residue of ss asprosin vs time. (**e**) Rg (Radius of gyration) vs time.

The secondary and tertiary structure of ss asprosin showed the presence of β-strand. In the present study, MD simulation of the 3D structure of ss asprosin protein was carried out for the refinement and conformational dynamics. RMSD analysis showed that the model attained a stable plateau after 85 ns with an average value of 0.95 nm. Fluctuation of Cɑ atoms of every amino acid during energy minimization assessed through RMSF showed no major fluctuations. However, residues at position 5-20 and 115-125 fluctuated with RMSF value of approximately 1 nm. During the initial time period between 1 and 10 ns, Rg pattern was unstable, but after 90 ns simulation time, Rg value showed stable behaviour with an average value of ~ 1.73 nm. The Ramachandran plot for geometry evaluation of ss asprosin protein after MD simulation using PROCHECK module revealed that 81.5% residues lie in the most favoured region, 17.7% residues in additional allowed region, 0.8% residues in generously allowed region and 0% residues in disallowed region (Fig. 5).

## Discussion

Since the discovery of asprosin in 2016^1^, there are at present no reports on asprosin in any of the other vertebrate groups. This is surprising as it is shown to play a critical role in metabolism, immunity and reproduction in mammalian species^1,6,13–16^. In the current study, the partial cDNA sequence for spotted snakehead *fbn1* and *furin* was obtained. ss *fbn1* comprises a 8454 bp long coding sequence that is predicted to code for 2817 amino acid long profibrillin protein with conserved furin cleavage site. The ss *furin* transcript retrieved from the transcriptome of *C. punctata* had 1869 bp ORF. The constitutive and ubiquitous expression of ss *fbn1* was observed in many tissues of *C. punctata*. Unlike humans, in which the maximum expression of *FBN1* has been reported in the white adipose tissue^1^, the gonads of *C. punctata* expressed the maximum *fbn1*. The gonads also show prominent gene expression of the protease *furin*, thereby indicating the potential production of asprosin in teleosts. Similar expression of *FBN1* and *FURIN* was also observed in the ovaries of beef heifers wherein it has been suggested to play an important role in ovarian functions^13^. The importance of asprosin in regulation of gonadal activities is further corroborated by a recent report in mice where Leydig and Sertoli cells are found to be immune-positive for asprosin and intratesticular administration of asprosin has been shown to promote steroidogenesis as well as spermatogenesis^17^. Based on our observations, and studies in mammals, it can be hypothesized that asprosin might be implicated in reproduction in teleosts also.

For the first time in a teleostean model, we have successfully cloned the *fbn1* region encoding asprosin protein of *C. punctata* and expressed it in the bacterial system. The IPTG-induced recombinant ss asprosin protein observed in the supernatant had an approximate molecular weight of 19 kDa. Intriguingly, the recombinant protein was not detected in the pellet which might be due to either the absence, or extremely low amount of inclusion bodies formed of recombinant ss asprosin. Recombinant ss asprosin was validated using anti-His antibody. In the case of human, the constructed recombinant protein has molecular weight of ~ 17 kDa with six histidine residues, however the molecular weight of asprosin found in human plasma is 30 kDa^1^. The discrepancy in size has been attributed to the lack of several post-translational changes in the recombinant asprosin expressed in the bacterial system as opposed to the asprosin found in the plasma. Indeed, it was shown that when recombinant asprosin is expressed in mammalian cell line, then the molecular weight is almost similar to that found in plasma^1^. Based on this observation, it can be suggested that the molecular weight of asprosin in *Channa punctata* might be more than 19 kDa and needs to be confirmed in further studies.

The percent identity matrix revealed that putative ss asprosin is more than 55% similar with the human asprosin and comprises 142 amino acids, whereas mammalian asprosin consists of 140 residues^1^. Interestingly, alignment of ss asprosin with human asprosin reveals that two amino acids at position 13th (Met) and 94th (Ser) exist in ss asprosin but are lost in due course of evolution in human. In silico analysis revealed that the putative ss asprosin and human asprosin were stable as its instability index, a parameter to measure the stability of protein under ex vivo conditions, were lower than the threshold value of 40^18^. The predicted ss asprosin and human asprosin had a high aliphatic index, thereby indicating its thermostability. The aliphatic index represents the relative volume of the aliphatic side chain (valine, alanine, leucine and isoleucine) occupied in a protein and determines the thermostability of a globular protein with values ranging between 71.13 and 143.54. Higher aliphatic value denotes a thermostable protein^19^. In addition to this, based on the negative grand average of hydropathicity index (GRAVY), the deduced ss asprosin and human asprosin seems to be hydrophilic in nature. The negative GRAVY value represents that a protein is hydrophilic and globular in nature, while positive GRAVY value implies a hydrophobic and membrane-bound protein^20^. The hydrophilic nature of ss asprosin implies that it is a soluble protein and might not require binding protein for its transport.

Post-translational analysis revealed the presence of glycosylation sites. Glycosylation is reported to increase the stability, as well as, half-life of proteins^21,22^. In case of other glycoproteins such as thyroid stimulating hormone, luteinizing hormone and follicle-stimulating hormone, glycosylation in these protein hormones helps in their interaction with the cognate receptor by increasing binding affinity and effecting the signalling^23–28^. The N-linked glycosylation sites in ss asprosin at position 3 and 37 correspond to the glycosylation sites in human asprosin^1^ and hence seem to be well conserved and might play a crucial role in the functional aspect of asprosin. Moreover, in ss asprosin, six phosphorylation sites are also predicted. Although phosphorylation plays a critical role in proteins involved in cell signalling, the implication of phosphorylation sites in ss asprosin is yet to be understood.

Currently, lack of NMR/crystal experimental structure of asprosin is a major constraint in understanding the structural aspect of the protein. This study provides the first comprehensive description of structural parameters using in silico approach via prediction of tertiary structure of asprosin in *C*. *punctata*. The presence of β-strands in the predicted 3D model of ss asprosin might be involved in strengthening the backbone of the protein and thereby enhancing the stability^29^. Similarly, tertiary structure of human asprosin predicted using bioinformatics tools also showed the presence of several β-strands^30^. Molecular dynamics (MD) simulation and energy minimization represent an important tool for the optimization of 3D models and relaxation of geometric chains with the unfavourable bond angle, bond length and torsion angles^31,32^. MD simulation of 3D model of ss asprosin was carried out for 100 ns and stability parameters revealed that it is stable in the physiological system. The RMSD value determines the protein stability on the basis of conformational changes in the Cα backbone of complete structure from the initial to the final position. The conformation of a protein is relative to the fluctuations occurring during the simulation process. These fluctuations are plotted as RMSD value vs the time during which simulation was in process. The smaller deviations in RMSD values indicate a stable structure^33^. After 80 s of simulation, the ss asprosin showed stable RMSD value of approximately 0.95 nm. RMSF value determines the fluctuations in the Cα atom of individual residue during MD simulation. The amino acid residues between position 5-20 and 115-125 in the 3D model of ss asprosin include coils and hence show more fluctuations in the RMSF value. Residues involved in the formation of loops, coils and turns and therefore more exposed to solvents are known to have high fluctuations in the RMSF value^34^. Loops, coils and turns are flexible random structures in the model and play crucial roles in protein function and folding^35,36^. The ss asprosin showed stable Rg value which is an indicator of globularity and compactness of the protein^37^. Further, the Ramachandran plot analysis revealed more than 80% residues of ss asprosin in the most favoured region and 0% residues in disallowed regions. Thus, the geometrical evaluation of ss asprosin along with RMSD, RMSF and Rg value attests the robustness of the predicted 3D model of putative ss asprosin protein.

## Conclusion

Asprosin is an important metabolic hormone, playing diverse roles in mammals and implicated in various metabolic disorders. The hormone is encoded by the C-terminal of the *FBN1* gene. Interestingly, since its discovery in 2016, there have been no studies on asprosin in any other vertebrate group. Since, teleost are being the most abundant extant vertebrates, we undertook the current study to explore the possibility of the presence of asprosin in a teleost model, *Channa punctata*. The expression of *fbn1* gene and the enzyme furin responsible for cleavage of the profibrillin into fibrillin-1 and asprosin, in various tissues of *C. punctata* suggest the presence of asprosin in teleost. The conserved furin cleavage site in the profibrillin helped in determining the putative primary ss asprosin sequence. Understanding the physicochemical properties of ss asprosin helped in determining the nature of the putative protein. Although, N-linked glycosylation sites in ss asprosin were found similar to human asprosin, the exact role of glycosylation and phosphorylation in asprosin are yet to be elucidated. The cloning and expression of ss recombinant asprosin has been carried out. In future, the purified ss recombinant asprosin could be used to understand the role played by this hormone in fish physiology, especially in reproductive functions.

## Material and methods

### Identification and validation of *fbn1* and *furin* gene transcript

The transcript of *fbn1* and *furin* gene were retrieved from the testicular transcriptome of *Channa punctata*, previously annotated with *Takifugu rubripes*, *Oreochromis niloticus* and *Rattus norvegicus* reference protein sequences^38^. The transcript with longest open reading frame (ORF) and maximum percentage identity was selected from several *fbn1* and *furin* transcripts with alternate ORF (Gene runner version 3.05, Hastings Software, Inc., USA) and using Blastx (http://blast.ncbi.nlm.nih.gov/Blast.cgi), the nucleotide sequence was verified.

Reverse transcriptase polymerase chain reaction (RT-PCR) was used to validate the transcript encoded sequence. Using Clustal omega, multiple sequence alignment was constructed to determine the conserved region for designing gene specific primers for *fbn1* and *furin* (http://www.ebi.ac.uk/Tools/msa/clustalo)^39^. Table 2 includes the details of the primers. For RT-PCR procedure, the following steps were employed: initial denaturation (95 °C for 5 min), 35 cycles of denaturation (95 °C for 30 s), annealing (60 °C for 30 s) and extension (72 °C for 45 s), followed by final extension (72 °C for 10 min). 1% agarose gel (Himedia, India) was used to resolve the amplified product of desired length with ethidium bromide staining. For elution, Wizard SV Gel and PCR Clean-Up System (Gel extraction kit Cat. No. A9281, Promega, USA) was used and Sanger sequencing was employed for *fbn1* and *furin* nucleotide sequence. Using Clustal Omega, the obtained partial sequence was aligned with the transcript encoded sequence and it exhibited complete similarity. The same partial sequence of *fbn1* and *furin* were submitted to the NCBI GenBank and accession number OP271666 and OP921042 has been assigned.

**Table 2:** Primers used for the reverse transcriptase Polymerase Chain reaction (RT-PCR) and qPCR.

S. no.	Types of primer		Primer sequence	Length
1	*fbn1* (Semi-quantitative)	Forward (5′-3′)	CTTGGTGGGTGGATACAGGT	20
		Reverse (5′-3′)	GTCTTTGTCATGTCTGTCCTCC	22
2	*fbn1* (qPCR)	Forward (5′-3′)	CCAAAGAAAGGACGCAAACG	20
		Reverse (5′-3′)	TCCTCGACATCCACACTG	18
3	*furin* (Semi-quantitative)	Forward (5′-3′)	GTAGGACGCAGAGTGAGT	18
		Reverse (5′-3′)	CTGAGGGGATTTTCGTTGG	19

### Expression analysis of *fbn1* and *furin* gene in *C. punctata*

#### Fish procurement and maintenance

Adult spotted snakehead *C. punctata* weighing 80–100 g was obtained from the wild populations in and around the National Capital Region of Delhi, India and stocked in dechlorinated fresh water tanks (15 fish per tank containing 45 L water) having dimension: 74 cm × 34 cm × 32 cm (L × B × H) under light regimen of 12 L:12 D at 25 ± 2 °C. The water was changed on alternate days and the water temperature was maintained at 24–26 °C. The fish were acclimatized for 3 weeks. The protocol has been approved by the Institutional Animal Ethics Committee (DU/ZOOL/IAEC-R/2021/6), Department of Zoology, University of Delhi and experiment was carried out following the relevant guidelines and regulations of the IAEC. The studies involving the live animals follows the recommendations in the ARRIVE guidelines.

#### Tissue-specific expression of *fbn1* and *furin*

Healthy spotted snakeheads (N = 6) were sacrificed with an overdose of 2-phenoxyethanol (5 mL/L water, Sisco Research Laboratories, Mumbai, India). Different tissues including brain (forebrain, hindbrain and midbrain), heart, liver, adipose tissue, gut, gonads and lymphoid organs including head kidney, spleen, skin, gills and trunk kidney were dissected out and stored at − 80 °C until RNA extraction after washing with 1×PBS.

#### RNA extraction and cDNA synthesis

Using the TRIzol reagent, total RNA was extracted from tissues following the manufacturer's protocol (Invitrogen). For assessment of RNA integrity, 1% agarose gel was used to observe the band intensities of 28S and 18S rRNA and RNA levels were quantified using a Nanodrop (ND-1000, Nanodrop Technologies, USA). The RNA samples having 1.8–2.0 absorbance ratio at A260/280 were selected for the cDNA preparation. In order to remove genomic contamination, RNA was treated with the enzyme DNase I (Thermo Scientific, USA) in 0.2 mL PCR tubes in a standard thermocycler at 37 °C for 30 min, and to terminate the DNase activity, EDTA (Thermo Scientific) was added and reaction was run at 65 °C for 10 min and then 4 °C hold. Using avian myeloblastosis virus (AMV) reverse transcriptase, the treated samples were processed for reverse transcriptional synthesis of single-strand cDNA following manufacturer’s specifications (Cat. No. K1622, Thermo Scientific, USA) in a thermocycler at 65 °C (for 10 min), 25 °C (for 5 min), 42 °C (for 60 min), 72 °C (for 10 min) and 4 °C hold. Through amplification of the 18S rRNA housekeeping gene, the synthesis of cDNA was confirmed.

#### Real-time quantitative PCR (qPCR) for *fbn1*

The partially validated sequence of *fbn1* was used for qPCR primers design in order to quantitate the target mRNA transcript. For validation of primers, melt curve analysis was employed. 1% agarose was used to resolve the amplified products of *fbn1* gene and using ethidium bromide staining method, single bands were visualized. The qPCR primers percentage efficiency was checked by a standard curve using two-fold serial dilutions of ovarian cDNA. Similarly, using specific primers, *18S* *rRNA* was quantified as a reference gene in each sample. The reaction was carried out in qPCR machine having standard cycle mode: 50 °C (for 2 min), 95 °C (for 2 min), 95 °C (for 15 s), 59 °C (for 15 s) and 72 °C (for 1 min) using power SYBR Green (Cat. No. 4367659, Applied Biosystems, USA) and finally a dissociation step: 95 °C (for 15 s), 60 °C (for 1 min) and 95 °C (for 15 s) for melt-curve analysis.

#### Semi-quantitative PCR (RT-PCR) for *furin*

Tissues expressing high levels of *fbn1* such as different parts of the brain (forebrain, midbrain and hindbrain), heart, testis and ovary were selected for studying the expression of *furin*. Using semi-quantitative primers of *furin* and *18S* *rRNA*, RT-PCR procedure was employed with the following steps: initial denaturation (95 °C for 5 min), 35 cycles of denaturation (95 °C for 30 s), annealing (60 °C for 30 s) and extension (72 °C for 45 s), followed by final extension (72 °C for 10 min). 1% agarose gel (Himedia, India) was used to resolve the amplified products of desired length with ethidium bromide staining. The gel was observed under ChemiDocTM XRS + Imaging system (Bio-rad).

#### Statistical analysis

*18S rRNA* expression was used to normalize the relative expression of *fbn1*. In order to calculate the relative fold change, 2^− ∆∆CT^ method^40^ was employed, wherein the tissue that showed lowest expression, i.e., head kidney was considered as a reference for tissue distribution. For the statistical analysis GraphPad Prism 8.0.1 software (GraphPad Software, La Jolla, CA) was used.

### Identification of furin cleavage site

For the prediction and analysis of putative peptide sequence encoded by *fbn1* gene, ExPASy server (http://ca.expasy.org/)^41^ was used. The sequence alignment was constructed for the putative partial sequence of profibrillin of *C. punctata* with the profibrillin of *Homo sapiens* using Clustal omega. Furin cleavage site (R-X-K/R-R↓ or R/K-X-X-X-K/R-R↓; where ↓ represents cleavage site)^2^ was manually identified in the aligned sequences near the C-terminal region. The amino acid sequence towards the C-terminus after the furin cleavage site represented the putative ss asprosin protein sequence as mentioned by the Romere et al.^1^.

### Molecular cloning and characterization of putative ss asprosin protein

#### Cloning

The C-terminal region of ss *fbn1* that is predicted to code for asprosin (8026–8454 bp) was cloned in the pProEx-HTc vector, having N-terminal His-tag. To clone the gene region of interest, the sequences of forward and reverse primers were 5′-CCTGGATCCTAAGCACTAACGCAACACACGATGAGC-3′ (carrying a BamHI site) and 5′-CGCAAGCTTTTAATGGAGGATGATCTGCACCCTC-3′ (containing a HindIII site). The amplified product using these primers was digested with the BamHI and HindIII restriction enzymes and the resulting fragment was ligated into the pProEx-HTc plasmid, which was also previously digested with the same restriction enzymes. After ligation, the plasmid containing gene of interest was transformed into the DH5α cells and grown overnight on 100 µg/ml ampicillin resistance agar plates at 37 °C. The white colonies from the plate were picked, grown and maintained in the LB medium supplemented with 100 µg/ml ampicillin at 37 °C overnight with constant shaking (220 rpm). The DH5α cells containing plasmid were pellet down and DNA was extracted using WizardⓇ Plus Minipreps DNA purification system (Cat. No. A7660, Promega, USA). The integrity of plasmid construct was validated through Sanger sequencing.

#### Expression

BL21(DE3) cells were transformed with pProEx-HTc vector derivative expressing asprosin protein and inoculated into LB medium containing 25 µg/ml chloramphenicol and 50 µg/ml ampicillin followed by incubation at 37 °C with constant shaking (220 rpm) until A_600_ reached at OD 0.6. 1 mM Isopropyl 1-thio-d-galactopyranoside (IPTG) was added in the culture for the induction of recombinant protein and culture was grown for an additional 2 h at 37 °C with constant shaking (220 rpm). The cells were pellet down and resuspended in cell lysis buffer containing 50 mM Tris–Cl, pH 8.0, 300 mM NaCl, 1 mM dithiothreitol, 1 mM EDTA, 10 mg/ml lysozyme, 0.1% SDS, 1× protease inhibitor cocktail and 1 mM phenylmethylsulfonyl fluoride and lysed by sonication. The cell lysate was then centrifuged at 12,000 rpm at 4 °C for 30 min. The supernatant and pellet were collected separately and stored at − 80 °C. Further, to check the induction of asprosin protein, protein samples from induced and uninduced cells were mixed with 2× SDS loading dye and loaded on 15% SDS-PAGE for gel electrophoresis and observed using coomassie brilliant blue staining. To validate the recombinant asprosin protein, western blotting was done using anti-His antibody (Anti-PolyHistidine-Peroxidase antibody, mouse monoclonal, Cat No. A7058, Sigma-Aldrich). The uninduced supernatant sample was taken as control. Briefly, for western blotting, protein was resolved in the 15% SDS-PAGE and transferred onto nitrocellulose membrane in transfer buffer (193 mM glycine, 25 mM Tris and 20% methanol, pH 8.5). After transfer, blocking of nitrocellulose membrane was done in 5% BSA for 1 h and subsequently incubated in the primary anti-His antibody (dilution 1:2000) for 1 h. The membrane was washed three times with TBST (50 mM Tris, 150 mM NaCl, 0.1% Tween-20, pH 7.6) and bands were developed using Luminata™ Crescendo Western HRP substrate (Millipore Corporation) and the luminescent image analyzer amersham imager-600 (GE/Biosciences AB) was used for visualization.

#### Physicochemical properties and post-translational modifications

To determine the molecular weight, grand average of hydropathicity index (GRAVY) and other physicochemical properties, the deduced primary sequence of asprosin of *C. punctata* and *H. sapiens* asprosin were subjected to ProtParam tool (http://expasy.org/cgi-in/protparam)^42^. Clustal omega was employed to compute the percentage similarity of deduced asprosin of *C. punctata* with asprosin of *H. sapiens*. Motifscan software (https://myhits.sib.swiss/cgi-bin/motif_scan) was employed for determining the post-translational modifications such as glycosylation and phosphorylation in the deduced asprosin of *C. punctata*.

#### Modelling and MD simulation

Automated modelling program I-TASSER (Iterative-Threading ASSEmbly Refinement) server^43^ was employed for modelling 3D structure of putative ss asprosin protein in order to gain insight into asprosin protein. For validation of the obtained 3D model, MD simulation was carried out using GROMACS (Groningen Machine for Chemical Simulations) 2019.2 version for 100 ns with force field Charmm27^44^. Simulation system was solvated with the TIP4P water model in a triclinic box. 25 Na^+^ and 15 Cl^−^ ions were added to the system for achievement of electroneutrality by steepest descent energy minimization. For the position restrained simulation, system equilibrium was done in two phases: NVT (constant number of particles, volume and temperature) and NPT (constant number of particles, pressure and temperature) ensemble for 500 ns each. System temperature and pressure was maintained at 300 K and 1 bar during MD runs via Berendsen and Parinello-Rahman methods. For long-range electrostatic interactions and bond length constrain, Particle Mesh Ewald (PME) and LINC algorithm were used^45^. GROMACS modules such as gmx rms, gmx rmsf and rmx gyrate were performed for protein analysis. After MD simulation, the 3D model of asprosin was subjected to the SAVES (Structural Analysis and Verification Server) and geometry evaluation was done using PROCHECK module (http://servicesn.mbi.ucla.edu/SAVES/)^46^.

### Ethics declaration

The protocol has been approved by the Institutional Animal Ethics Committee (DU/ZOOL/IAEC-R/2021/6), Department of Zoology, University of Delhi and all the methods were performed in accordance with the relevant guidelines and regulations of the IAEC. The studies involving the live animals follows the recommendations in the ARRIVE guidelines.

## Supplementary Information

Supplementary Figure S1.

Supplementary Figure S2.

Supplementary Figure S3.

## Data Availability

The datasets analysed during the current study are available in the National Centre for Biotechnology Information (NCBI) GenBank repository [Accession number OP271666 and OP921042].
