# Supplementary figures and images for: Molecular cloning, characterization and 3D modelling of spotted snakehead fbn1 C-terminal region encoding asprosin and expression analysis of fbn1
Source: Sci Rep. 2023 Mar 18;13:4470. doi: 10.1038/s41598-023-31271-x (PMC10024713; doi:10.1038/s41598-023-31271-x)

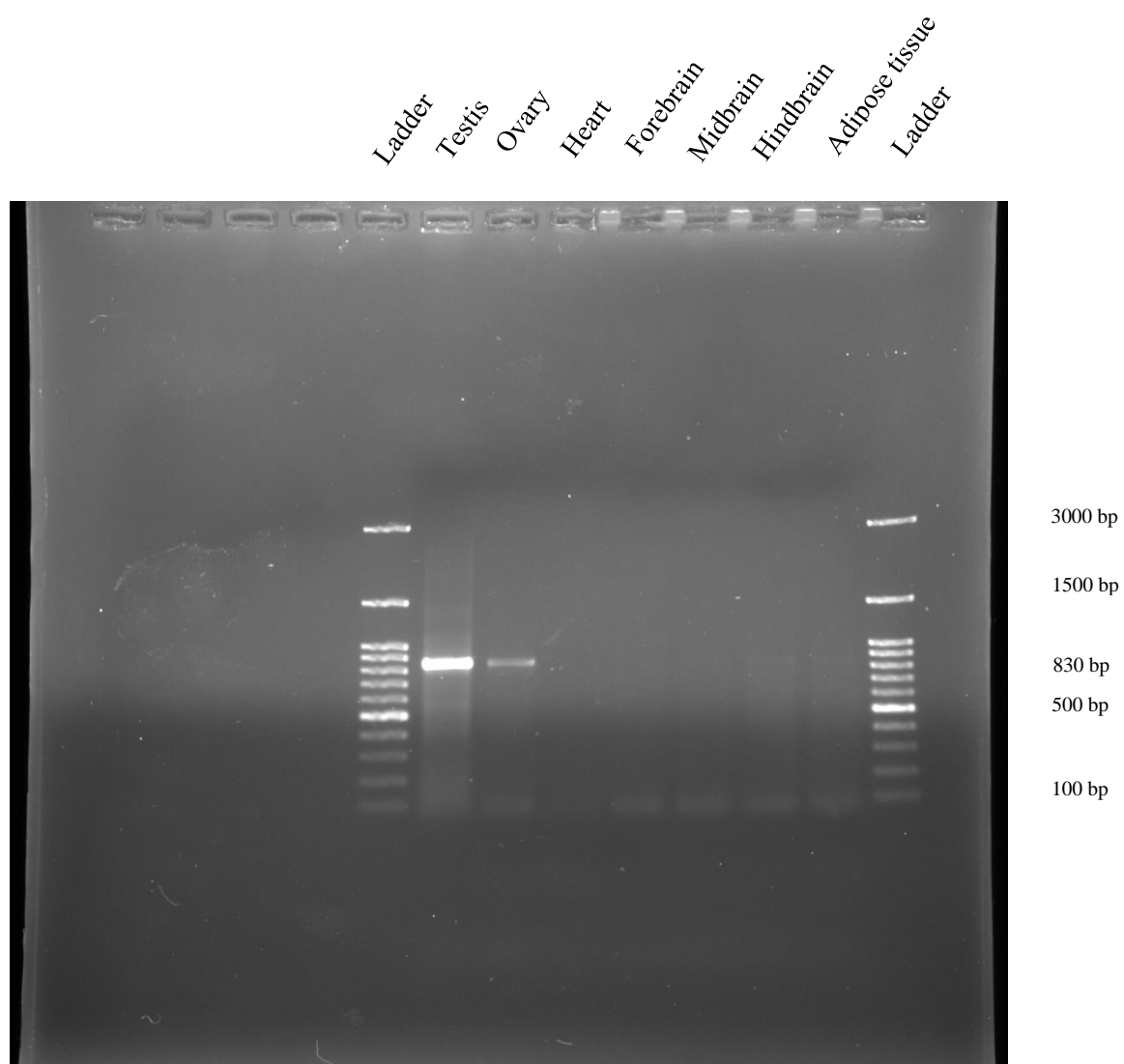

**Supplementary Figure S1:** Tissue distribution of *furin* using semi quantitative PCR.

Supplement: Supplementary file 1 — Supplementary Figure S1. [file 41598_2023_31271_MOESM1_ESM.pdf]
