# Supplementary material for: Molecular cloning, characterization and 3D modelling of spotted snakehead fbn1 C-terminal region encoding asprosin and expression analysis of fbn1
Source: Sci Rep. 2023 Mar 18;13:4470. doi: 10.1038/s41598-023-31271-x (PMC10024713; doi:10.1038/s41598-023-31271-x)
Supplement: Supplementary file 2 — Supplementary Figure S2. [file 41598_2023_31271_MOESM2_ESM.pdf]

MSYYHHHHHHDYDIPTTENLYFQGAMGILSTNATHDEPVDDMQLTENEMISLASVD
VEDTLEFHLNISDLSNRDHILEFTPALSTLSDHVRYSIDYGNEEGYFKINQREGVSYLH
LSKKKSLSPGAYYLQISSVPLYRKKELAELEDRHDKDYLTGQLGDILKRRVQIILH

Supplementary Figure S2: Recombinant asprosin (Yellow highlighted region is asprosin sequence)
