# Supplementary material for: Molecular cloning, characterization and 3D modelling of spotted snakehead fbn1 C-terminal region encoding asprosin and expression analysis of fbn1
Source: Sci Rep. 2023 Mar 18;13:4470. doi: 10.1038/s41598-023-31271-x (PMC10024713; doi:10.1038/s41598-023-31271-x)
Supplement: Supplementary file 3 — Supplementary Figure S3. [file 41598_2023_31271_MOESM3_ESM.pdf]

(a) (b) (c)

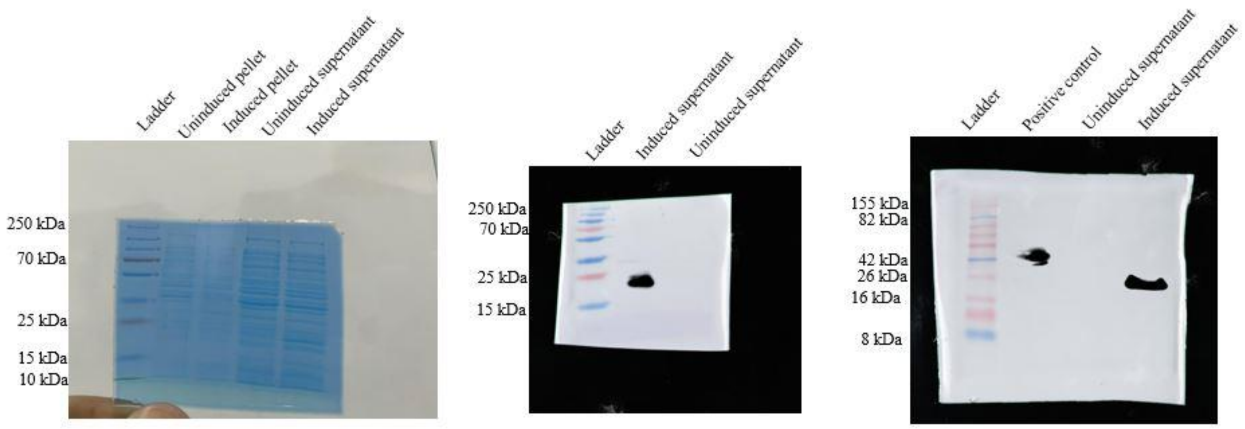

Expression analysis of recombinant ss asprosin

(a) 15% SDS-PAGE showing the protein expression in the uninduced pellet, induced pellet, uninduced supernatant and induced supernatant samples observed using coomassie staining.

(b) Western blotting using anti-His antibody for validation of recombinant asprosin in supernatant of induced and uninduced samples.

(c) Western Botting using anti-His antibody for validation of recombinant asprosin in supernatant of induced and uninduced samples with histidine tag protein as positive control.
